# Investigating Patient Use and Experience of Online Appointment Booking in Primary Care: Mixed Methods Study

**DOI:** 10.2196/51931

**Published:** 2024-07-08

**Authors:** Helen Atherton, Abi Eccles, Leon Poltawski, Jeremy Dale, John Campbell, Gary Abel

**Affiliations:** 1 Primary Care Research Centre School of Primary Care, Population Science, and Medical Education University of Southampton Southampton United Kingdom; 2 Unit of Academic Primary Care Warwick Medical School University of Warwick Coventry United Kingdom; 3 University of Exeter Medical School Exeter United Kingdom

**Keywords:** appointment, patient appointments, online systems, primary health care, general practice, qualitative research, secondary data analysis, mobile phone

## Abstract

**Background:**

Online appointment booking is a commonly used tool in several industries. There is limited evidence about the benefits and challenges of using online appointment booking in health care settings. Potential benefits include convenience and the ability to track appointments, although some groups of patients may find it harder to engage with online appointment booking. We sought to understand how patients in England used and experienced online appointment booking.

**Objective:**

This study aims to describe and compare the characteristics of patients in relation to their use of online appointment booking in general practice and investigate patients’ views regarding online appointment booking arrangements.

**Methods:**

This was a mixed methods study set in English general practice comprising a retrospective analysis of the General Practice Patient Survey (GPPS) and semistructured interviews with patients. Data used in the retrospective analysis comprised responses to the 2018 and 2019 GPPS analyzed using mixed-effects logistic regression. Semistructured interviews with purposively sampled patients from 11 general practices in England explored experiences of and views on online appointment booking. Framework analysis was used to allow for comparison with the findings of the retrospective analysis.

**Results:**

The retrospective analysis included 1,327,693 GPPS responders (2018-2019 combined). We conducted 43 interviews with patients with a variety of experiences and awareness of online appointment booking; of these 43 patients, 6 (14%) were from ethnic minority groups. In the retrospective analysis, more patients were aware that online appointment booking was available (581,224/1,288,341, 45.11%) than had experience using it (203,184/1,301,694, 15.61%). There were deprivation gradients for awareness and use and a substantial decline in both awareness and use in patients aged >75 years. For interview participants, age and life stage were factors influencing experiences and perceptions, working patients valued convenience, and older patients preferred to use the telephone. Patients with long-term conditions were more aware of (odds ratio [OR] 1.43, 95% CI 1.41-1.44) and more likely to use (OR 1.65, 95% CI 1.63-1.67) online appointment booking. Interview participants with long-term conditions described online appointment booking as useful for routine nonurgent appointments. Patients in deprived areas were clustered in practices with low awareness and use of online appointment booking among GPPS respondents (OR for use 0.65, 95% CI 0.64-0.67). Other key findings included the influence of the availability of appointments online and differences in the registration process for accessing online booking.

**Conclusions:**

Whether and how patients engage with online appointment booking is influenced by the practice with which they are registered, whether they live with long-term conditions, and their deprivation status. These factors should be considered in designing and implementing online appointment booking and have implications for patient engagement with the wider range of online services offered in general practice.

## Introduction

### Background

Booking an appointment or service online is widespread internationally, with most sectors, including travel, entertainment, and hospitality, offering this facility. Health care has adopted online appointment booking with varying levels of patient uptake and engagement [1-3]. In England, most primary care is delivered by National Health Service (NHS) general practices to registered lists of patients. All NHS general practices must make appointments available for online booking [4,5], with appointments booked via web platform or app. This is part of the contract that general practices have with NHS England, the body responsible for leading the NHS in England. Despite the availability of online appointment booking being mandatory since 2012, uptake by patients has been slow and variable, with national-level data showing that, in March 2023, a total of 43% of patients were registered to use an online appointment booking service [6].

The use of online appointment booking is intended to lead to reduction in reception staff workload and increased patient satisfaction. It can offer flexibility, convenience, and time saving [7-13] and allow carers of older adults to make and track health care appointments [14]. It may reduce the likelihood of patients missing appointments [11]. It is known that people who use online appointment booking tend to be female, educated to degree level, and more frequent users of the internet [9,10,15,16]; use is lower in non-White patients, in lower socioeconomic groups, and in those with poorer health [9,10,15,16]. Patients from medically underserved and vulnerable populations are less likely than other patient groups to access and use online health technologies [17]. Specific barriers to engagement with online services for these groups include a lack of experience, knowledge, and skills when using the internet [17-19]; lack of technical support; and lower health literacy [17,20].

The routine use of online services in general practice settings, such as online triage platforms and video consultation [21-23], increased during the COVID-19 pandemic. Understanding who benefits and who does not benefit from the use of online appointment booking and why is important given the potential for inequality of access to primary care services to be exacerbated when services move online [19]. As an online service with a defined purpose and broad geographic availability, online appointment booking potentially provides a marker for how patients may engage with the wider range of online services within modern primary care. Evidence that seeks to understand how patients interact with online health care services is needed to shape such services to suit patients and their needs as we adjust to a new normal for primary care delivery. With levels of uptake of online appointment booking low in general practice and a lack of evidence on why this is the case, we conducted this mixed methods study.

### Objectives

We sought to describe and compare the characteristics of patients in relation to their use of online appointment booking to explore how patients’ use relates to their experience of care and investigate patients’ views on online appointment booking arrangements in general practice.

## Methods

### Approach

We conducted a retrospective analysis of data on awareness and use of online appointment booking from the annual national General Practice Patient Survey (GPPS) conducted in English general practices. This was complemented by a semistructured interview study with patients from a range of general practices in England with differing levels of uptake of online appointment booking. The interviews were conducted both before and during the COVID-19 pandemic.

In UK general practice, patients can book an appointment online directly with the general practice they are registered with. Patients can do this using the NHS website, app, or via a web platform with app option provided by their general practice. General practices use a web platform often with app option, of their own choosing to offer online appointment booking, and there are multiple suppliers of these systems in the UK market. Patients can use these services via an internet-enabled device (eg, smartphone, tablet, or computer) according to their preference. These systems are not necessarily linked to the electronic health record, which is also chosen by the general practice from a range of available systems. This study examined online appointment booking across all platform and associated app types, and we did not set out to compare platforms as all offer online appointment booking in line with NHS requirements.

### Retrospective Analysis of GPPS

#### Data Source

We conducted secondary analyses of data from the English GPPS [24]. The GPPS is a postal and online survey of patients’ experience of primary care in England. The survey is sent annually (January-March) to approximately 2.2 million adult patients, with the findings published each July [25]. A stratified sample of patients aged >16 years is drawn from the practice list of each general practice in England. Patients from practices known from previous surveys to have low response rates are oversampled to ensure an adequate number of responses per practice. We used data from the 2018 and 2019 surveys making use of 2 questions, one asking about awareness of online services in the respondents’ general practice and another asking about their use of online services in the previous 12 months. These were used in conjunction with data from the survey on demographic characteristics and health status [26].

#### Analysis

Descriptive analyses and logistic regression were used to investigate the percentage of patients who reported awareness of their general practice offering online appointment booking and recent use of online appointment booking. These analyses were restricted to patients reporting trying to make an appointment at their general practice in the last 12 months. This is important because patients may not have used online appointment booking (or been aware of the option) due to not needing an appointment.

Multivariate logistic regression was used to examine associations between both awareness and use of online appointment booking and age, gender, ethnicity, deprivation (based on the Index of Multiple Deprivation [IMD] [27] corresponding to the respondents’ postcode of residence), presence of a long-term condition, long-term sickness, and being deaf.

Comparison of models accounting and not accounting for clustering by practice (using a random effect) was used to illustrate the extent to which disparities reflect the clustering of certain types of patients in practices where awareness and use of online appointment booking is high or low for all patient groups. The random effect from these models was used to quantify the variability in the odds of patients being aware of or using online appointment booking between practices.

Further models augmented those described previously to include other GPPS report and evaluation items (as predictors of online appointment booking awareness and use) regarding how easy practice websites were to use, whether respondents had a preferred general practitioner (GP), how helpful receptionists were, ease of getting through on the phone, and use of online prescription ordering. A final set of regression models examined the extent to which awareness and use of online appointment booking were associated with patients’ experiences of making an appointment, choice of appointment, ability to see their preferred GP, and their overall experience. These models were adjusted for the same patient factors included in the previous models as well as including a random effect for practice.

Full statistical details are provided in Multimedia Appendix 1. Analysis was conducted using Stata (version 12; StataCorp).

### Semistructured Interview Study

#### Sampling and Recruitment

Interview participants were recruited from 11 general practices selected for maximum variation in levels of online booking, list size, rurality, deprivation, and ethnic diversity. There were three recruitment waves: (1) a scoping survey was distributed to patients to gather data on their demographic characteristics (gender, age, educational level, employment status, health status, and ethnicity), booking behaviors and awareness of online booking, and willingness to participate in an interview; (2) interview volunteers were purposively sampled according to background and booking behavior and invited to take part in an interview; and (3) finally, aiming to fill demographic gaps within the sample, potential participants were identified based on their background via general practices and invited directly to interview. Patients who were aged <18 years or at end of life, lacked capacity to provide informed consent, or had not recently booked a general practice appointment were excluded from participation. The COVID-19 pandemic meant that recruitment was paused as the funder made the decision to halt all research activity not related to COVID-19, allowing practices to focus on their COVID-19 response. Recruitment was resumed in June 2020, at which time we made use of direct invites to interviews (wave 3 detailed previously) to make up the remainder of the sample.

#### Data Collection

A total of 43 interviews were carried out via telephone, audio recorded, transcribed, and anonymized.

During the interviews, we explored in-depth patients’ experiences of and views on booking appointments online, including how they did this and, for those who had not used it, whether they knew about it or not. The topic guide can be found in Multimedia Appendix 2.

#### Data Analysis

We used the framework method to conduct a comparative thematic analysis of the interview data relevant to the key findings from the retrospective analysis of GPPS data. The framework method is commonly used in health research in which research questions are relevant to policy. It has 5 key stages: familiarization, identifying a thematic framework, indexing, charting, and mapping and interpretation [28]. A matrix was used with “cases” arranged along one axis and “codes” along the other. This allowed data to be organized systematically to compare differing experiences and perspectives on key areas from participants’ accounts. The NVivo software (version 12; QSR International) was used to support this process.

### Patient and Public Involvement

We involved members of the public at various stages of the study. A lay coapplicant joined the study team and attended meetings, provided advice throughout, and helped facilitate workshops. Public workshops were held to refine our protocol (before funding was awarded), gain input on the participant-facing documentation, and discuss the implications of the study findings. We piloted the scoping survey and telephone interviews with 3 contributors. In total, 2 contributors also provided their interpretation of the qualitative data.

### Ethical Considerations

We obtained NHS research ethics approval and Health Research Authority approval for the semistructured interview study from the West Midlands – Solihull Research Ethics Committee (study reference: 19/WM/0272). The secondary analysis of GPPS data did not require ethics or Health Research Authority approval.

Respondents to the GPPS are anonymous with their identifying data not collected at initial data collection. In the information provided with the survey, potential respondents are informed that their responses may be shared with NHS approved researchers. Providing a response to the survey is taken as implied consent to agree to the conditions laid out in the information provided.

Informed consent was obtained from all participants in the semistructured interview study.

Interview transcripts were deidentified, allocated an identifying code and stored separately from participant information to ensure anonymity. All data were stored in password protected secure electronic storage. Participants in the semistructured interview study were given a GBP £10 (US $12.7) shopping voucher as a thank you for the time taken in participating. Interview quotes are used with consent, and basic demographic information is supplied with the quotes for context.

## Results

### Retrospective Analysis of GPPS

#### Overview

Of the 1,327,693 GPPS responders (2018-2019 combined) who reported attempting to make an appointment within the previous 12 months, 45.11% (581,224/1,288,341) were aware that their practice offered online appointment booking, and 15.61% (203,184/1,301,694) reported using online appointment booking. We found very strong evidence (*P*<.001) of associations between both awareness and use of online appointment booking and all variables considered in both unadjusted and adjusted models except for the effect of rurality in the adjusted model, including a random effect for practice when considering awareness (Tables 1 and 2).

There were only small differences between the unadjusted and adjusted models, with consistent direction of association except for awareness among patients aged 25 to 44 years and for rural versus urban location (Tables 1 and 2). Findings from the adjusted model included a random effect for practice and so can be interpreted as differences experienced by patients registered with the same general practice.

**Table 1:** Awareness of online booking of general practice appointments (N=1,288,341).

Variable			Frequency^a^				Unadjusted^b^, OR^c^ (95% CI)		Adjusted^b^, OR (95% CI)		Adjusted+RE^b,d^, OR (95% CI)
			Total respondents, n (%)		Respondents aware of online booking (n=581,224), n (%)						
**Sex**											
	Male	587,137 (45.57)		245,470 (41.81)		Reference		Reference		Reference	
	Female	688,801 (53.46)		331,432 (48.12)		1.24 (1.23-1.25)		1.25 (1.24-1.26)		1.27 (1.26-1.28)	
**Age group (y)**											
	16-24	122,879 (9.54)		50,573 (41.16)		0.75 (0.73-0.76)		0.89 (0.87-0.90)		0.86 (0.84-0.88)	
	25-34	208,897 (16.21)		95,574 (45.75)		0.94 (0.93-0.95)		1.13 (1.11-1.15)		1.10 (1.08-1.12)	
	35-44	214,993 (16.69)		99,001 (46.05)		0.96 (0.95-0.97)		1.13 (1.11-1.15)		1.09 (1.08-1.11)	
	45-54	230,452 (17.89)		108,454 (47.06)		1.00 (0.99-1.01)		1.11 (1.09-1.12)		1.09 (1.08-1.10)	
	55-64	203,277 (15.78)		99,884 (49.14)		1.07 (1.06-1.08)		1.13 (1.12-1.14)		1.13 (1.12-1.15)	
	65-74	168,014 (13.04)		80,836 (48.11)		Reference		Reference		Reference	
	75-84	94,412 (7.33)		34,188 (36.21)		0.62 (0.62-0.63)		0.61 (0.60-0.62)		0.58 (0.57-0.59)	
	≥85	34,431 (2.67)		8915 (25.89)		0.40 (0.39-0.41)		0.38 (0.37-0.39)		0.34 (0.33-0.35)	
**IMD^e^ quintile**											
	1—least deprived	249,352 (19.35)		128,348 (51.47)		Reference		Reference		Reference	
	2	253,343 (19.66)		121,617 (48.00)		0.87 (0.86-0.88)		0.87 (0.86-0.88)		0.91 (0.90-0.92)	
	3	256,313 (19.89)		117,260 (45.75)		0.78 (0.77-0.78)		0.77 (0.77-0.78)		0.85 (0.84-0.86)	
	4	262,693 (20.39)		113,058 (43.04)		0.69 (0.68-0.69)		0.69 (0.68-0.69)		0.77 (0.76-0.78)	
	5—most deprived	265,750 (20.63)		100,578 (37.85)		0.54 (0.53-0.55)		0.54 (0.53-0.55)		0.66 (0.65-0.67)	
**Rurality**											
	Urban	1,112,590 (86.36)		502,382 (45.15)		Reference		Reference		Reference	
	Rural	175,751 (13.64)		78,843 (44.86)		1.08 (1.07-1.09)		0.96 (0.95-0.97)		1.01 (0.96-1.06)	
**Ethnic group**											
	Asian	107,325 (8.33)		44,346 (41.32)		0.81 (0.80-0.82)		0.87 (0.86-0.89)		0.89 (0.88-0.91)	
	Black	41,896 (3.25)		16,085 (38.39)		0.73 (0.71-0.74)		0.83 (0.81-0.85)		0.79 (0.77-0.81)	
	Mixed	19,296 (1.5)		8,557 (44.35)		0.94 (0.91-0.97)		0.96 (0.93-1.00)		0.91 (0.88-0.95)	
	White	1,076,912 (83.59)		497,261 (46.17)		Reference		Reference		Reference	
	Other	24,836 (1.93)		8306 (33.44)		0.59 (0.57-0.60)		0.65 (0.63-0.68)		0.63 (0.61-0.65)	
**Long-term condition**											
	Yes	689,896 (53.55)		328,180 (47.57)		1.15 (1.15-1.62)		1.38 (1.36-1.39)		1.43 (1.41-1.44)	
	No	531,919 (41.29)		228,690 (42.99)		Reference		Reference		Reference	
**Occupation**											
	Other	1,187,020 (92.14)		540,342 (45.52)		Reference		Reference		Reference	
	Sick, disability, or both	50,585 (3.93)		21,152 (41.81)		0.84 (0.82-0.85)		0.80 (0.78-0.81)		0.80 (0.79-0.82)	
**Deafness and sign language use**											
	Yes	5508 (0.43)		1933 (35.09)		0.65 (0.62-0.69)		0.78 (0.73-0.84)		0.76 (0.71-0.81)	
	No	1,266,842 (98.33)		573,583 (45.28)		Reference		Reference		Reference	
**Period**											
	2018	641,337 (49.78)		277,392 (43.25)		Reference		Reference		Reference	
	2019	647,004 (50.22)		303,832 (46.69)		1.17 (1.16-1.18)		1.18 (1.17-1.19)		1.19 (1.18-1.20)	

^a^Frequency numbers are weighted and rounded. Regression results are not weighted.

^b^*P* values from joint Wald test were computed for the adjusted and unadjusted models. All *P* values are <.001 except that corresponding to rurality in the mixed-effects logistic regression model.

^c^OR: odds ratio.

^d^RE: random effect.

^e^IMD: Index of Multiple Deprivation.

**Table 2:** Variation in use of online booking of general practice appointments (N=1,301,694).

Variable			Frequency^a^				Unadjusted^b^, OR^c^ (95% CI)		Adjusted^b^, OR (95% CI)		Adjusted+RE^b,d^, OR (95% CI)
			Total respondents, n (%)		Respondents aware of online booking (n=203,184), n (%)						
**Sex**											
	Male	592,522 (45.52)		89,505 (15.11)		Reference		Reference		Reference	
	Female	696,669 (53.52)		112,135 (16.1)		1.04 (1.03-1.05)		1.04 (1.03-1.05)		1.04 (1.02-1.05)	
**Age group** **(y)**											
	16-24	124,337 (9.55)		17,548 (14.11)		0.94 (0.91-0.96)		1.14 (1.11-1.18)		1.13 (1.09-1.16)	
	25-34	211,051 (16.21)		36,138 (17.12)		1.21 (1.18-1.23)		1.52 (1.49-1.55)		1.48 (1.45-1.51)	
	35-44	217,280 (16.69)		37,319 (17.18)		1.22 (1.20-1.24)		1.47 (1.44-1.49)		1.43 (1.40-1.46)	
	45-54	232,622 (17.87)		39,142 (16.83)		1.19 (1.17-1.21)		1.34 (1.32-1.37)		1.34 (1.31-1.36)	
	55-64	205,150 (15.76)		34,604 (16.87)		1.16 (1.14-1.18)		1.23 (1.21-1.25)		1.24 (1.22-1.26)	
	65-74	169,731 (13.04)		25,833 (15.22)		Reference		Reference		Reference	
	75-84	95,635 (7.35)		9003 (9.41)		0.59 (0.58-0.60)		0.57 (0.56-0.59)		0.56 (0.55-0.57)	
	≥85	34,840 (2.68)		2217 (6.36)		0.41 (0.40-0.43)		0.37 (0.36-0.39)		0.35 (0.34-0.37)	
**IMD^e^ quintile**											
	1—least deprived	251,435 (19.32)		45,936 (18.27)		Reference		Reference		Reference	
	2	255,837 (19.65)		42,270 (16.52)		0.88 (0.87-0.90)		0.87 (0.86-0.89)		0.90 (0.89-0.92)	
	3	258,902 (19.89)		39,996 (15.45)		0.79 (0.78-0.81)		0.77 (0.76-0.79)		0.83 (0.81-0.84)	
	4	265,735 (20.41)		39,569 (14.89)		0.75 (0.74-0.76)		0.70 (0.69-0.72)		0.75 (0.74-0.77)	
	5—most deprived	268,897 (20.73)		35,270 (13.12)		0.62 (0.61-0.63)		0.57 (0.56-0.58)		0.65 (0.64-0.67)	
**Rurality**											
	Urban	1,124,219 (86.37)		178,247 (15.86)		Reference		Reference		Reference	
	Rural	177,475 (13.63)		24,937 (14.05)		0.97 (0.95-0.98)		0.91 (0.90-0.93)		0.92 (0.88-0.97)	
**Ethnic group**											
	Asian	108,829 (8.36)		18,500 (17.00)		1.10 (1.08-1.12)		1.11 (1.08-1.13)		1.02 (1.00-1.05)	
	Black	42,387 (3.26)		5666 (13.37)		0.83 (0.81-0.86)		0.89 (0.86-0.92)		0.76 (0.73-0.78)	
	Mixed	19,511 (1.5)		3,475 (17.81)		1.19 (1.14-1.25)		1.15 (1.10-1.21)		1.03 (0.98-1.09)	
	White	1,087,517 (83.55)		169,378 (15.57)		Reference		Reference		Reference	
	Other	25,204 (1.94)		3777 (14.99)		1.00 (0.96-1.04)		1.02 (0.97-1.06)		0.92 (0.87-0.96)	
**Long-term condition**											
	Yes	697,247 (53.56)		120,347 (17.26)		1.24 (1.23-1.25)		1.59 (1.57-1.61)		1.65 (1.63-1.67)	
	No	537,166 (41.27)		74,212 (13.82)		Reference		Reference		Reference	
**Occupation**											
	Other	1,198,858 (92.1)		189,142 (15.78)		Reference		Reference		Reference	
	Sick, disability, or both	51,258 (3.94)		7646 (14.92)		0.94 (0.92-0.97)		0.85 (0.82-0.87)		0.86 (0.84-0.89)	
**Deafness and sign language use**											
	Yes	5611 (0.43)		1046 (18.64)		1.18 (1.10-1.27)		1.23 (1.13-1.33)		1.21 (1.11-1.32)	
	No	1,280,135 (98.34)		200,171 (15.64)		Reference		Reference		Reference	
**Period**											
	2018	647,454 (49.74)		93,728 (14.48)		Reference		Reference		Reference	
	2019	654,240 (50.26)		109,456 (16.73)		1.18 (1.17-1.20)		1.19 (1.18-1.20)		1.19 (1.18-1.21)	

^a^Frequency numbers are weighted and rounded. Regression results are not weighted.

^b^*P* values from joint Wald test were computed for the adjusted and unadjusted models. All *P* values are <.001.

^c^OR: odds ratio.

^d^RE: random effect.

^e^IMD: Index of Multiple Deprivation.

#### Age

**Figure 1:**
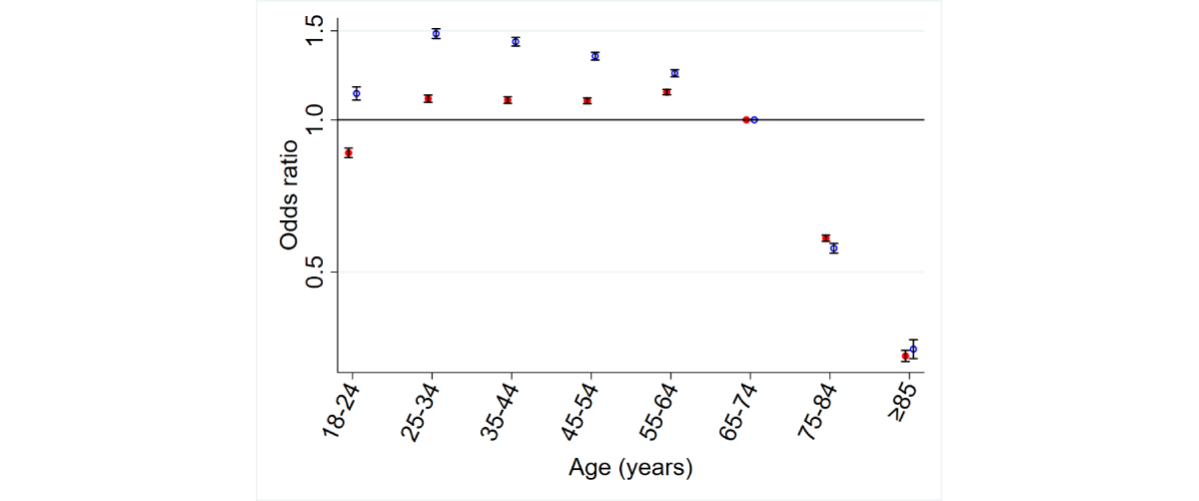
Age and awareness and use of online appointment booking. OR: odds ratio.

There was little variability in both awareness and use among those aged <65 years but a substantial drop in both for those aged >75 years (odds ratio [OR] for awareness [those aged ≥85 years vs 65-74 years] 0.34, 95% CI 0.33-0.37; Tables 1 and 2 and Figure 1).

#### Deprivation Status

**Figure 2:**
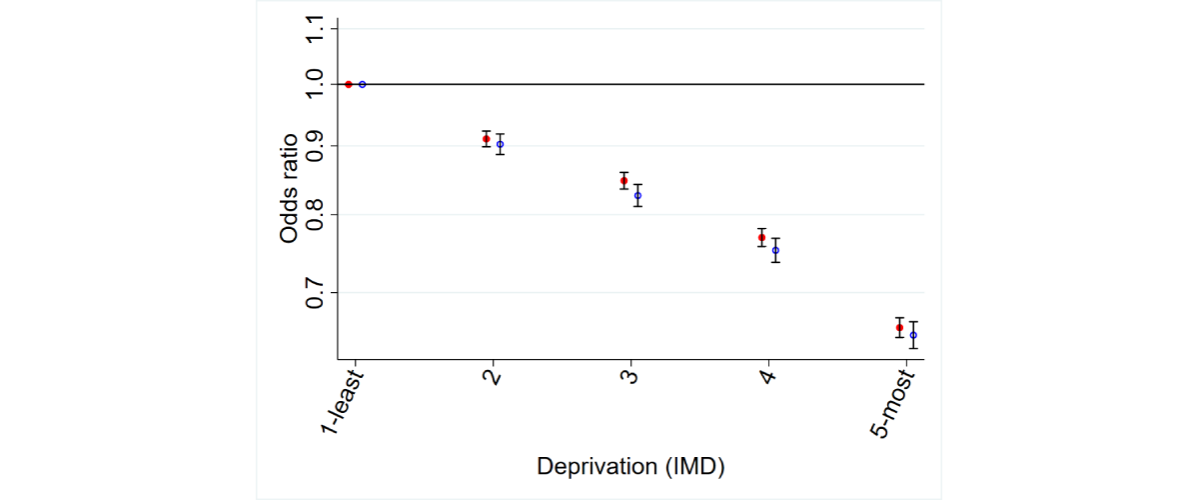
Deprivation and awareness and use of online appointment booking. IMD: Index of Multiple Deprivation.

There was a strong deprivation gradient, with more deprived patients being less likely to use or be aware of online appointment booking (OR for use [most vs least deprived] 0.65, 95% CI 0.64-0.67; Tables 1 and 2 and Figure 2).

#### Ethnicity

**Figure 3:**
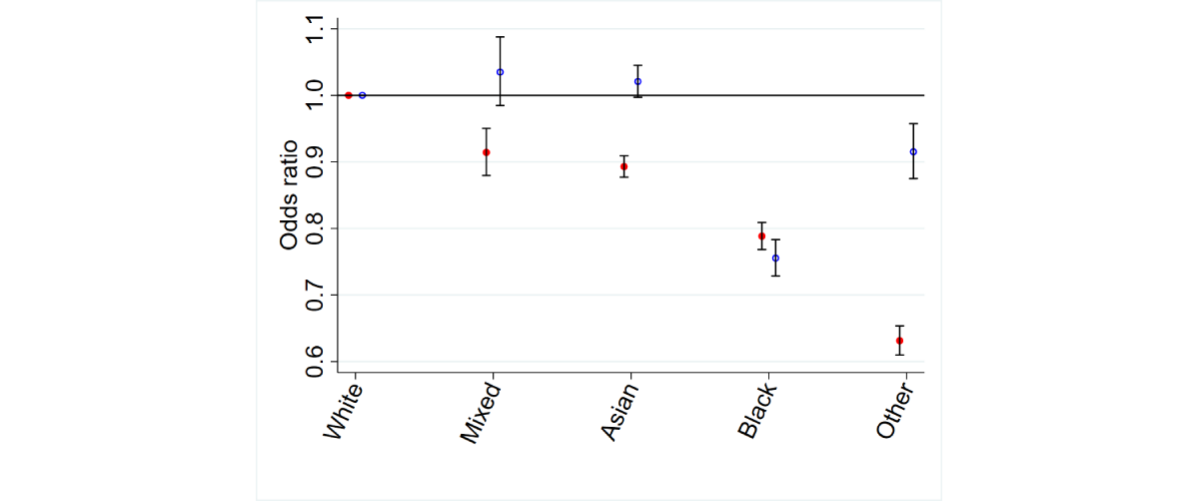
Ethnicity and awareness and use of online appointment booking.

A more complex relationship was observed for ethnicity. All patients from ethnic minority groups were less likely to be aware of online appointment booking than White patients. However, only patients in the Black and *Other* ethnic groups were less likely to use online appointment booking than White patients, and mixed and Asian patients were somewhat more likely to use online appointment booking (Tables 1 and 2 and Figure 3).

#### Location

**Figure 4:**
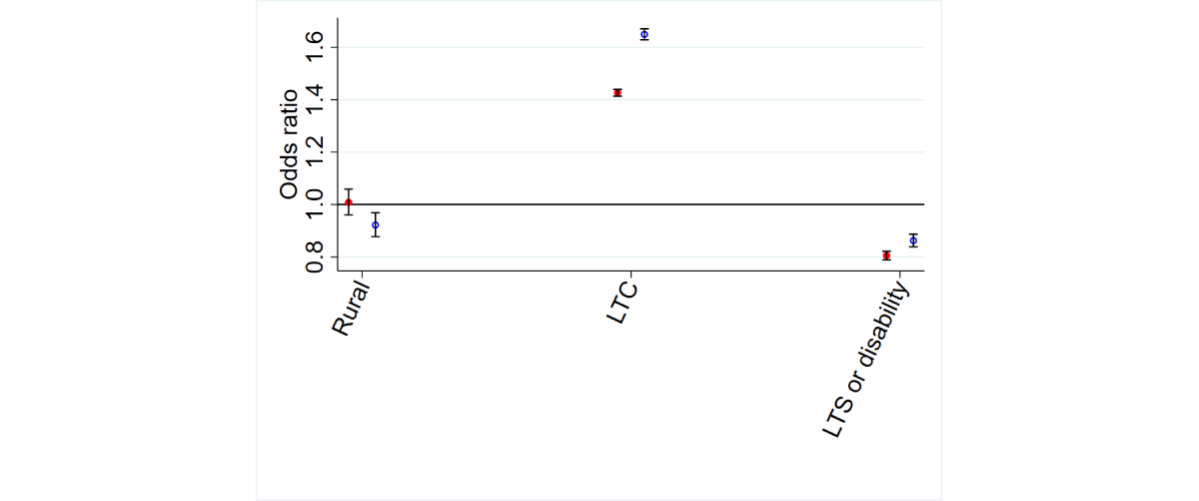
Location and long-term conditions (LTCs) and awareness, and use, of online appointment booking. LTS: long-term sickness.

Patients living in rural areas were less likely to be aware of or use online appointment booking than patients living in urban areas (OR for use [rural vs urban] 0.92, 95% CI 0.88-0.97; Tables 1 and 2 and Figure 4).

#### Health Status

Patients who were permanently sick or had a disability were less likely to be aware of or use online appointment booking than those living in urban areas and patients who were not sick or patients without disabilities (OR for awareness [sick or disability vs not sick or no disability] 0.80, 95% CI 0.78-0.82; Tables 1 and 2 and Figure 4).

Patients with long-term conditions were more likely to be aware of and use online appointment booking than patients without them (OR for awareness [long-term condition vs no long-term condition] 1.43, 95% CI 1.41-1.44; Tables 1 and 2 and Figure 4). Interestingly, patients who were deaf and used sign language were less likely to be aware of but more likely to use online appointment booking than those without such an impairment (OR for use [deaf vs not deaf] 1.21, 95% CI 1.11-1.32).

#### Year

Comparing the GPPS data from the 2018 and 2019 surveys, both awareness and use increased across this period (OR for awareness in 2019 vs 2018, 1.19, 95% CI 1.18-1.20; OR for use in 2019 vs 2018, 1.19, 95% CI 1.18-1.21).

#### Practice

When comparing models that did and did not account for the patients’ registered practice, the patient groups that were less likely to use or be aware of online appointment booking remained the same between models (except for rural patients’ awareness, where the effect was small in both cases). The strongest factor in determining awareness and use of online appointment booking was the practice the patient was registered with. For all factors apart from deprivation, the changes in OR magnitudes were small (Tables 1 and 2). The deprivation gradient in awareness and use of online appointment booking was noticeably stronger in models that did not incorporate adjustment for practice, which implies that patients living in deprived areas are more likely to be registered at practices where awareness and use is low for all patients (Tables 1 and 2).

The OR covering 95% of practices (comparing the highest with the lowest) estimated from the random-effects models was 18.1 (95% CI 17.2-19.0) for awareness and 16.7 (95% CI 15.8-17.7) for use. These are considerably larger than the ORs associated with any patient factor, suggesting that the practice a patient was registered with was the strongest predictor of awareness and use of online appointment booking when compared with a wide range of other potentially important variables.

#### Patient Experience

Patients reporting their practice website being easy to use were far more likely to be aware of and use online appointment booking (OR for use [“Very easy to use” vs “Not easy at all”] 6.34, 95% CI 6.06-6.64; Table 3). Having a preferred GP was associated with somewhat higher awareness and use, whereas ease of getting through to a practice on the phone was associated with lower use and somewhat lower awareness. Patients who reported finding practice receptionists helpful were more likely to be aware of online booking but less likely to use it. Patients who had ordered repeat prescriptions online were also more likely to be aware of and use online appointment booking than those who had not (OR 3.58, 95% CI 3.50-3.62).

**Table 3:** Odds ratio (OR) for awareness and use of online booking adjusted for demographic factors, presence of long-term condition, occupation, deafness, and period and for clustering of patients within practices.

Variable			Awareness				Use		
			OR (95% CI)		*P* value^a^		OR (95% CI)		*P* value^a^
**Have you ordered repeat prescriptions online?**					<.001				<.001
	No	Reference				Reference			
	Yes	1.84 (1.80-1.87)				3.58 (3.50-3.62)			
**How easy is it to get through to someone at your GP^b^ on the phone?**					<.001				<.001
	Very easy	0.85 (0.82-0.88)				0.63 (0.61-0.65)			
	Fairly easy	0.97 (0.94-1.00)				0.80 (0.77-0.82)			
	Not very easy	0.99 (0.96-1.02)				0.90 (0.87-0.93)			
	Not at all easy	Reference				Reference			
**How helpful do you find the receptionist at your GP?**					<.001				<.001
	Very helpful	1.15 (1.09-1.21)				0.72 (0.68-0.76)			
	Fairly helpful	1.12 (1.06-1.18)				0.83 (0.79-0.88)			
	Not very helpful	1.03 (0.97-1.08)				0.91 (0.86-0.97)			
	Not at all helpful	Reference				Reference			
**Is there a particular general practitioner you usually prefer to see or speak to?**					<.001				<.001
	Yes, for all appointments	1.19 (1.16-1.21)				1.61 (1.58-1.64)			
	Yes, for some appointments	1.29 (1.26-1.32)				1.56 (1.53-1.59)			
	No	Reference				Reference			
**How easy is it to use your GP website?**					<.001				<.001
	Very easy	4.34 (4.19-4.51)				6.34 (6.06-6.64)			
	Fairly easy	2.77 (2.68-2.86)				3.68 (3.52-3.85)			
	Not very easy	1.58 (1.53-1.64)				1.81 (1.73-1.90)			
	Not easy at all	Reference				Reference			

^a^*P* values from joint Wald tests.

^b^GP: general practice.

A final set of models (Table 4) showed that, regardless of whether patients had tried online booking, those who were aware of it were more likely than patients who were not aware of it to have a positive overall experience of their general practice, see or speak with their preferred GP, be offered a choice of appointment, or report a positive overall experience of making an appointment. Furthermore, patients who were aware of but had not used online appointment booking were more likely than unaware patients to be satisfied with their appointment type. Surprisingly, patients who were aware of and used online appointment booking were less likely than patients who were not aware of it to be satisfied with their appointment type.

**Table 4:** Odds ratio (OR) for different outcome measures adjusted for demographic factors, presence of long-term condition, occupation, deafness, and period and for clustering of patients within practices.

Awareness and use of online appointment booking		OR (95% CI)	*P* value^a^	
**Outcome—overall experience of general practice^b^**				<.001
	Unaware	Reference		
	Aware but not used	1.34 (1.32-1.36)		
	Aware and used	1.27 (1.25-1.30)		
**Outcome—see or speak with preferred GP^c,d^**				<.001
	Unaware	Reference		
	Aware but not used	1.25 (1.23-1.27)		
	Aware and used	1.43 (1.40-1.45)		
**Outcome—offered a choice of appointment^e^**				<.001
	Unaware	Reference		
	Aware but not used	1.17 (1.16-1.18)		
	Aware and used	1.42 (1.40-1.44)		
**Outcome—satisfied with appointment type^f^**				<.001
	Unaware	Reference		
	Aware but not used	1.16 (1.14-1.17)		
	Aware and used	0.95 (0.93-0.96)		
**Outcome—overall experience of making an appointment^g^**				<.001
	Unaware	Reference		
	Aware but not used	1.22 (1.20-1.23)		
	Aware and used	1.07 (1.06-1.09)		

^a^*P* values from joint Wald tests.

^b^Overall, how would you describe your experience of your general practice?

^c^GP: general practitioner.

^d^How often do you see or speak to your preferred GP when you would like to?

^e^On this occasion, were you offered a choice of appointment?

^f^Were you satisfied with the type of appointment you were offered?

^g^Overall, how would you describe your experience of making an appointment?

### Semistructured Interview Study

#### Overview

We conducted 43 semistructured qualitative interviews with individuals who had recently booked general practice appointments (Tables 5 and 6). Participants were aged 18 to ≥85 years and had a range of educational levels and employment statuses. In total, 47% (20/43) of the participants reported having at least one long-term condition. Of the 43 participants, 18 (42%) had tried online appointment booking, 16 (37%) were aware that they could access online appointment booking but had not tried it, and 9 (21%) were unaware that online appointment booking was available.

**Table 5:** Characteristics of participating patients (N=43).

Characteristic					Participants, n (%)
**Sex**					
	Male			22 (51)	
	Female			21 (49)	
**Age** **(y)**					
	18-24			2 (5)	
	25-34			5 (12)	
	35-44			6 (14)	
	45-54			5 (12)	
	55-64			9 (21)	
	65-74			6 (14)	
	75-84			6 (14)	
	≥85			3 (7)	
**Educational level**					
	None			2 (5)	
	Secondary education			6 (14)	
	Further education (16-18 years)			4 (9)	
	Higher education (18+ years)			24 (56)	
	Other or do not know			5 (12)	
**Employment status**					
	Full time			13 (30)	
	Part time			3 (7)	
	Student			2 (5)	
	Unemployed			1 (2)	
	Sick, disability, or both			5 (12)	
	Retired			11 (26)	
	Home or family			2 (5)	
	Other			5 (12)	
**Condition**					
	Long-term condition or conditions			20 (47)	
	No long-term condition			6 (14)	
	Unknown			1 (2)	
**Ethnicity**					
	White—English, Welsh, Scottish, Northern Irish, or British			34 (79)	
	White other			3 (7)	
	Other ethnic group			6 (14)	
**Experience with online booking**					
	**Awareness**				
		Tried online booking	18 (42)		
		Aware but not tried online booking	16 (37)		
		Unaware	9 (21)		
	**Use**				
		Never use online booking	29 (67)		
		Sometimes use online booking	3 (7)		
		Frequently use online booking	11 (26)		

**Table 6:** Demographic details on participating general practices in the interview study (N=11).

			Practices, n (%)
**Online booking use**			
	High	4 (36)	
	Medium	6 (55)	
	Low	1 (9)	
Rural			3 (27)
Semirural			3 (27)
Urban			5 (45)
**List size**			
	Small	3 (27)	
	Medium	4 (36)	
	Large	4 (36)	
**IMD^a^**			
	Below average	6 (55)	
	Above average	5 (45)	
Coventry and Warwickshire			7 (64)
Devon			4 (36)

^a^IMD: Index of Multiple Deprivation.

The framework analysis identified 3 key areas in which there was comprehensive qualitative evidence that matched the data identified in the quantitative element of the study. These were (1) general practice–mediated factors, (2) impact of age and life stage, and (3) specific experiences of those with a long-term condition. Patients also shared information about their differing levels of confidence and experience using online services in general.

#### General Practice–Mediated Factors

How the general practice organized online appointment booking systems influenced patient experience.

Patients had varied awareness and understanding of the online appointment booking system available to them. Routes to learn about online appointment booking included advertisements on noticeboards, SMS text messages, letters, or being told directly by practice staff. Despite all participating practices having online appointment booking available, many participants were not aware of it and described not having seen or heard any information about it:

The first time I went the reception, what do you call it the receptionist? She turned round and said, “Well, you can also book online.”

Male participant; aged 55-64 years; practice 4; White British

I mean, you could be on hold for up to half an hour trying to call through. And the thing is, it’s never been advertised to us that you could book online. Like, I’ve never seen it anywhere or been informed in any communication that you can do it online, so that’s why that’s always been my way of booking it.

Male participant; aged 25-34 years; practice 4; White British

The practices varied in the processes that patients described having to follow to register for online appointment booking. The most common requirement was to attend the practice in person to collect a form to complete and then return with identification before they could be registered. In contrast, other participants stated that their practice had not required them to show any identification.

Providing identification required the patient to be proactive in seeking registration. There were several examples of participants describing an intention to register and then not completing the process:

I got as far as bringing, bringing the form home and filling it in. But then because I don’t go past the surgery on a daily basis I never took it, I never took it back.

Male participant; aged 55-64 years; practice 7; White British

There was also variation among general practices in the appointments made available to book online, with some patients describing that their practice appeared to make very few appointments available. A lack of appointments that could be booked urgently or soon was experienced as a key barrier to use in cases in which patients needed this kind of appointment:

Right, I have tried to book online...and I saw that there was the option. [...] And that thrill was met with disappointment when I saw the lack of slots available online.

Female participant; aged 25-34 years; practice 6; White European

Where participants found that they could book appointments on the same day or within the week, this was a key facilitator to using and continuing to use online appointment booking:

I much prefer it, it’s, it’s quite, quite easy to learn how to do it, it’s, is simple to use, it’s fast, and it’s reliable. [...] I’ve managed to get an appointment within a day or two sometimes.

Male participant; aged ≥85 years; practice 12; White British

Overall, online appointment booking was seen as fitting into a modern society, which relies heavily on online services:

It’s probably very, very good for some people, but it’s not for me. I appreciate that we are in a digital age and we, you know, we’re relying on these, the computer more, and online, and e-mails and stuff, but no, I’m, I really don’t think it is an advantage in some cases. It’s not for me.

Female participant; aged 65-74 years; practice 10; White Irish

Patients, including those who did not use online appointment booking, were accepting of its value in the context of it being available as one of several options for accessing an appointment in general practice. Across the sample, online appointment booking was seen as a tool that was a good fit for some patients in some circumstances:

Well, I think in this day and age most people can do it online [...] I think a range of all three, I mean, going forward I suppose with staffing and stuff, you know, they’re obviously looking to minimise the face-to-face contact, but I still think there’s a, a need for that at, at some, some level for everybody.

Female participant; aged 55-64 years; practice 12; White British

#### Age and Life Stage

The convenience of online appointment booking was particularly appealing for those in the workforce. Booking an appointment via telephone could be problematic due to having to call while at work or the lack of privacy in an employment setting:

If you’re at work then you, you need permission off your managers that you’re going to be on the phone, especially if you work in a very busy environment and there’s just no way you can just be on the phone for ages. You know, like, you might be Number 23 [...] in the, in the order, so you’re going to wait and wait, so, you know, sometimes it’s a bit difficult. [...] And then you, waiting, what, when you do get on the phone then they will ask you what the problem is and sometimes you don’t really want to say, it’s quite confidential and personal.

Female participant; aged 55-64 years; practice 4; Asian British

Online appointment booking was advantageous when childcare or caring responsibilities made it difficult for patients to call the general practice when telephone lines opened in the morning:

When she was at primary school and my eldest was at primary school, I wouldn’t, I wouldn’t be able to ring at half eight because I’d be in the car, or I’d be stood in the playground. You know, it, it doesn’t work.

Female participant; aged 45-54 years; practice 11; White British

Some patients regarded their age as leading to a reluctance to use online appointment booking. Online appointment booking was regarded as something new to learn that was unfamiliar. Some participants did not use the internet regularly, did not use smartphones to access such services, and saw switching on a computer as inconvenient:

People that are younger than me and just do this automatically online, you know, whereas I don’t do it automatically.

Female participant; aged 75-84 years; practice 12; White British

Requiring help to use online appointment booking was seen as disempowering. Older patients were accustomed to booking an appointment using the analog telephone and did not explore the possibility of using another route. Sometimes, using online appointment booking meant relying on family members or a partner to do the “internet admin” in whatever format this might take.

#### Differing Experiences of Those With a Long-Term Condition

Patients in the sample who had experience using online appointment booking and had long-term conditions found that the service was a good fit for them. Participants with long-term conditions who used online appointment booking said that they had only become aware of and subsequently used online appointment booking when accessing the online system to obtain their repeat prescriptions for their long-term condition:

I actually first used the app to get a repeat prescription [...] Then I noticed that you could also book appointments on there.

Female participant; aged 25-34 years; practice 4; White British

Accessing repeat prescriptions provided a gateway to use other online services, including online appointment booking, and this was also demonstrated in the quantitative findings.

Patients with long-term conditions were used to booking preplanned routine appointments for the management of their condition. Online appointment booking allowed them to do this at a time that was convenient and in a way that was convenient, be this via smartphone or computer:

It was for a regular, you know, prescription update thing, so it wasn’t like I needed it on the day, it was something I could plan.

Female participant; aged 35-44 years; practice 1; White British

This reflected the nonurgent nature of the appointments they were booking, which fit with how online appointment booking was being offered at their practice, where appointments could be more readily booked some time ahead than on the day.

## Discussion

### Principal Findings

Awareness of online appointment booking is much higher than use, although still under half of patients are aware of online appointment booking. Patients, including those who did not use it, were accepting of its value as a convenient option for accessing an appointment.

There was little variability in awareness and use among those aged <65 years but a substantial decline in those aged >75 years. Age and life stage were key factors influencing experiences and perceptions. Working patients and those with caring responsibilities found it particularly convenient relative to the telephone, but older patients in the interview sample preferred to use the telephone as it was familiar and did not require the help of others.

There was a strong deprivation gradient, with deprived patients being less likely to use or be aware of online appointment booking. Findings related to ethnicity were more complex, with all minority groups less likely to be aware of online appointment booking than White patients, whereas patients in the “Black” or “other” ethnic groups were less likely to use online appointment booking than White patients and those in the “mixed” or “Asian” ethnic groups were more likely to have used them. Location was influential, with patients in rural areas less likely to be aware of or use online appointment booking. While the interview study sampled patients from practices with a range of IMD scores (6/11, 55% of practices were below the average IMD), from different ethnic groups, and from a range of rural and urban areas, we did not observe any findings related directly to these factors.

Health status was a key factor. Those who were permanently sick or had a disability were less likely to be aware of or use online appointment booking. Patients with long-term conditions found that it was a good fit for them, being useful for booking routine nonurgent appointments for their condition. The only group where there was a disparity between awareness and use was in deaf patients who used sign language, who were less likely to be aware of but more likely to use online appointment booking. This was the only group in which there was such disparity between awareness and use (compared with the wider population), suggesting that, although deaf people may find online appointment booking particularly useful, they are unlikely to be aware that it is an option.

The strongest factor in determining awareness and use of online appointment booking across the study was the general practice the patient was registered with. The influence of general practice–mediated factors on patient experience was a key finding in the interview study. There was variation in how general practices organized their appointments and the registration process required for online appointment booking, both of which impacted experience.

Patient experience was a key focus of this study and impacted awareness and use of online appointment booking. Patients who found the practice website easy to use, patients with a preferred GP, and patients who had ordered repeat prescriptions online were more likely to be aware of and use online appointment booking. Patients reported that accessing repeat prescriptions was their gateway to use other online services, including online appointment booking. Patients reporting that it was easier to get through to the general practice on the phone had lower use and somewhat lower awareness, and patients reporting receptionists as helpful were more likely to be aware of but less likely to use online appointment booking. Regardless of whether they had tried it, patients who were aware of online appointment booking were more likely to have had a positive experience with their general practice when arranging and obtaining an appointment than those who were not aware of it.

### Limitations

Our study was conducted on data from the 2018 to 2019 GPPS, and much has changed in health care delivery since. However, the 2022 GPPS showed that the number of patients booking appointments online had only risen to 21%, representing a change of just 5% over the period since 2018 to 2019. The 2022 GPPS also asked how patients chose to book an appointment, and just 16% of patients booked online.

The GPPS is a large national survey; it includes responses from patients registered with every general practice in England, and provides a high generalizability of findings. Like similar surveys, the GPPS has a relatively modest response rate (34% in 2018 and 33% in 2019). While low response rates may lead to substantial biases in absolute percentages, they are less likely to result in biased estimates of associations [29].

The interview study was disrupted by the COVID-19 pandemic. Recruitment was paused for 3 months, when the funder made the decision to halt all research activity not related to COVID-19, allowing general practices to focus on their COVID-19 response. Interviews were conducted via telephone, and therefore, we could interview participants already recruited before mid-March 2020 and adhere to social distancing restrictions*.* Recruitment was resumed in June 2020, and we revised our approach (as outlined in the Methods section) using a purposive approach to ensure that our sample was as diverse as possible.

Online appointment booking was unavailable to patients from the onset of the COVID-19 pandemic, and general practices used telephone and online triage to control the availability of face-to-face appointments. Online appointment booking resumed later in 2020 and has been available since. This may have influenced the responses to the interviews that were conducted after the onset of the COVID-19 pandemic, although we were not able to identify anything specific related to this.

We aimed for a maximally variable sample in the interview study*.* This allowed us to provide qualitative findings contextualizing the quantitative results according to age and presence of long-term conditions. However, the proportion of participants from ethnic minority groups was slightly lower than those in the population of England and Wales in the 2021 census (14% in our sample vs 18.3% in England and Wales) and was not reflective of the populations of the included practices in all cases. Underrecruitment to health research studies of people from ethnic minority groups is a long-standing concern within the research community and can be attributed to researchers not facilitating access to research studies, with rapport with participants being a key factor in increasing recruitment [30,31]. This was a challenge during the COVID-19 pandemic. Further exploration of the views and experience of patients from ethnic minority groups is needed.

Our interview data did not provide insights into the impact of deprivation as identified in the retrospective analysis. This may be due to our sample, with a smaller number of interview participants having lower levels of education (a proxy for deprivation levels). The interview participants were not sampled for location (rural or urban) as we did not realize the significance of this distinction ahead of conducting the study. Future studies should consider these factors.

Our study was conducted in the United Kingdom, where there is a national health service and health care is free at the point of use. Findings may differ in countries where health systems differ, particularly those where primary care is accessed on a fee-for-service basis.

### Comparison With Prior Work

Our study included online appointment booking via any online interface for a general practice appointment. This included the NHS app, where appointments can be booked. The NHS app had 18 million registrations during the COVID-19 pandemic due to its use for vaccination passports [32]. A study examined the use of the NHS app between January 2019 and May 2021. Those researchers examined total number of downloads, registrations, prescriptions ordered, records accessed, and appointments booked. Using time-series analysis, they calculated that, 12 months after the first UK COVID-19 lockdown, there were 21,606 fewer appointments booked online than might have been expected if the COVID-19 lockdown had not occurred [33], indicating that the COVID-19 pandemic had a detrimental effect on the use of online appointment booking.

A US observational study examining a patient portal [34] offering online appointment booking found that use of the portal was low and use was lowest in persons of lower socioeconomic status and without broadband internet. Another observational study in the United States examined the use of patient portal functions by patients, including appointment booking [35]. As in our study, the study demonstrated that use of the appointment booking function decreased with age, with the sharpest decrease in those aged >65 years. A cross-sectional survey of general practice patients in the West Midlands, England, looked at use of online appointment booking services before the pandemic [16]. The study observed similar associations to those in our study between awareness and use and deprivation level and between awareness and use and long-term conditions. Our study reinforces these findings as well as adding explanations for some of the patterns identified within the qualitative findings. Patterns related to age and socioeconomic status, as well as ethnicity, which we could say less about, have been identified as structural barriers to the equitable use of digital health by those examining the political economy of digital health equity [36]. This has implications for those designing and devising online services in health care.

### Future Research

Experience with the general practice was a key factor in awareness and use of online appointment booking, and this warrants further research into exactly how practices can best enable patients to access and use online services and the different types of support that may be needed for different patient groups. General practices in England offer online services through portals that are provided by various third-party companies. Alongside these portals is the NHS app. Future research should explore how the different interfaces impact experience and use of online services and what that means for uptake. A survey exploring patient views on what supports uptake and use of web-based services in primary care found that there were varied factors impacting their use of these services, including poor design of online interfaces and lack of support from the general practice, along with personal factors such as preference for human interaction [37].

Older patients used online appointment booking less and, in some cases, found it difficult to use online services or chose not to when the telephone was available and they were allowed to book an appointment through it. Services may wish to focus on supporting those older people who do want to use online services while maintaining a telephone service for those who cannot or do not wish to use online routes. Research has demonstrated that understanding the characteristics of GPs, including their personalities, can influence the digital maturity levels in a given general practice, and so examining the GPs in a practice may contribute to a deeper understanding of how and why patients choose to use or not use online services [38].

Our qualitative interviews showed that using an online repeat prescription service was a gateway to using other online services. Researchers and those designing services may wish to explore why this service is relatively highly accessed when compared to other online services to determine what makes an online service usable. In the case of online appointment booking, accessing repeat prescriptions raised awareness of online appointment booking services, and we have demonstrated that most patients are not aware of this service.

Our quantitative findings identified that deaf patients who use sign language may find online appointment booking particularly useful but they are unlikely to be aware that it is an option. We did not recruit any deaf patients to the interview study, and their views are likely to be of importance in future research.

We identified a clear deprivation gradient, and this may exacerbate inequitable access for patients. The risk of excluding patients who cannot access online services should be a key consideration in the design of the wider-access systems within general practices and a key factor examined in future research. To ensure equitable access, it is important to understand how particular groups (patients who are older, more deprived, or from ethnic minority groups) who were shown to be less likely to be aware of and use online booking can be supported should they wish to use these services and not disadvantaged should they choose not to.

Patient awareness of online appointment booking may be influenced by the actions of the general practice and how they promote these services. A systematic review examining how primary care supports patients to become aware of and access online services highlighted the lack of evidence in this area but concluded that existing evidence indicated that there was the potential for such support to increase awareness and use of services, with more research needed to understand what this support would look like [39].

### Conclusions

Awareness and use of online appointment booking by patients in general practice is associated with the characteristics of the practice they attend, their age, the level of deprivation associated with their general practice, and whether they have a long-term condition. There are clear contextual explanations behind why online booking is more appealing for those with long-term conditions and those aged <75 years. Other factors such as why deprivation or ethnicity mediates use of online booking remain less understood. The findings have implications for patient engagement with the wider range of online services offered in general practice, how these are delivered, and for future research in this area.

## Appendix

Multimedia Appendix 1 Detailed statistical methods.

Multimedia Appendix 2 Interview topic guide.

## Abbreviations

GP: general practitioner
GPPS: General Practice Patient Survey
IMD: Index of Multiple Deprivation
NHS: National Health Service
OR: odds ratio
