# Supplementary material for: Investigating Patient Use and Experience of Online Appointment Booking in Primary Care: Mixed Methods Study
Source: J Med Internet Res. 2024 Jul 8;26:e51931. doi: 10.2196/51931 (PMC11263895; doi:10.2196/51931)
Supplement: Multimedia Appendix 1 [file jmir_v26i1e51931_app1.docx]

**Detailed statistical methods**

Data were analysed from the 2018 and 2019 English General Practice Patient Surveys (GPPS), separately and combined, and restricted to those who report attempting to make a general practice appointment in the last 12 months. The GPPS survey is a postal and online national survey covering different aspects of patients’ experience of primary care. It is sent to a stratified random sample of adult patients registered with an English primary care practice. There were 660,085 and 667,608 responses in 2018 and 2019 respectively (34.1% and 33.1% response rate respectively) and these were received from approximately 7,000 general practices. Full details of the survey development and methodology are published elsewhere.^1, 2^

Awareness and use of online booking of GP appointments was assessed by two questions (questions 4 and 5 on both questionnaires):

1. As far as you know, which of the following online services does your GP practice offer? *Booking appointments online, ordering repeat prescriptions online, accessing my medical records online, none of these, don’t know.*
2. Which of the following general practice online services have you used in the past 12 months? *(response as previous question)*

Comparisons of awareness and use of online booking was made between different patient groups based on sociodemographic and health status characteristics (age, gender, ethnicity, deprivation, the presence of long-term condition, not working due to long-term sickness/disability, being deaf and using sign language), collected through the GPPS.

Unadjusted and adjusted logistic regression models with awareness and use of online booking as binary outcomes were performed to assess crude associations and associations adjusted for all known and available confounding factors.

Survey non-respondent weights were used in all descriptive analyses to reduce bias in any estimates but were not included in the regression analyses as factors included in the weighting calculations were adjusted for in these models.

Subsequently, we extended the adjusted models to include random effects for practice. These models quantify the extent to which disparities reflect the clustering of certain types of patients in practices where online booking of appointment is well used as had been done previously with other aspects of patient experience.^3^ Or in other words, it might be the case that certain patient groups are registered in practices with general low awareness and use of online booking and that this is responsible for the overall low awareness and use in that group. In order to assess this, we compare models that do and do not account for the practice patients are registered at.

A further series of models further extended the mixed effects models to include other factors reported in the GPPS in order to examine their influence on awareness and use of online booking: patients having ordered repeat prescriptions online, ease of getting through on the phone, helpfulness of receptionist at the practice, patients having a preferred GP and ease of using the GP practice website.

A final set of regression models examined the extent to which awareness and use of online appointment booking was associated with patients’ experiences of making an appointment, choice of appointment, ability to see their preferred GP and their overall experience. These models adjusted for the same patient factors included in the previous models as well as including a random effect for practice.

Crucially, as mentioned above, all analyses were restricted to patients reporting trying to make an appointment at their GP surgery in the last 12 months. This is important because patients may not have used online booking (or been aware of the option) due to not needing an appointment.

1. Campbell J, Smith P, Nissen S, et al. The GP Patient Survey for use in primary care in the National Health Service in the UK – development and psychometric characteristics. *BMC Family Practice* 2009; 10: Doi: 10.1186/1471-2296-1110-1157.

2. NHS England. GP Patient Survey, <https://gp-patient.co.uk/surveys-and-reports> (accessed 05/03/2018).

3. Lyratzopoulos G, Elliott M, Barbiere JM, et al. Understanding ethnic and other socio-demographic differences in patient experience of primary care: evidence from the English General Practice Patient Survey. *BMJ Qual Saf* 2012; 21: 21-29. 2011/09/09. DOI: 10.1136/bmjqs-2011-000088.
