# Supplementary material for: Investigating Patient Use and Experience of Online Appointment Booking in Primary Care: Mixed Methods Study
Source: J Med Internet Res. 2024 Jul 8;26:e51931. doi: 10.2196/51931 (PMC11263895; doi:10.2196/51931)
Supplement: Multimedia Appendix 2 [file jmir_v26i1e51931_app2.docx]

**Interview guide**

INTRODUCTION
Our research team looks at GP services and we are currently working on a project that is specifically about online appointment booking for GP services. We are really interested in finding out more about what people think about the process of booking GP appointments and, views and experiences of doing this online (this may be via a website or app).

As researchers we are not for or against any particular way of booking appointments, but we would like to understand more about what people really think. We are interested in how people book appointments (whether it be online, by phone or in person), their experiences of using online appointment booking, and their views about such services.

Does that sound clear to you?

Is there anything you would like to ask at this stage?

[TAKE VERBAL CONSENT]

**PART ONE – How they book appointments**

To start this interview, I would like you to think back to the last GP appointment you made. Would you mind describing to me how you went about booking your appointment? And the reasons why you booked your appointment in this way? [Probe for further information behind reasoning if necessary]

Do you always book your GP appointments in this way? Or do you use other methods too? [Prompt: phone, in-person, online]. Can you tell me more about the times you have booked appointments in this way and reasons why?

Would the reason you are booking an appointment affect how you go about booking an appointment? [Prompt: urgency of problem, health professional, for someone else]

Would your circumstances affect how you go about booking an appointment? [at work, time of day, location]

**PART TWO (A) –** **Online appointment booking experiences (People who *HAVE* booked online)**

**Can you tell me about when you first used online appointment booking to make a GP appt?**

Prompts:

- How did use come about?
- How did you find out about it?
- What made you decide to use it?

**Can you tell me about your experience of using/trying to use online appointment booking?**

Prompts:

- How do you use it – phone, laptop, desktop?
- How long did it take you to get used to using it?
- How do you feel about using it? Why?
- Encounter any difficulties?
- How have these been addressed?

**How does it compare to other ways you can make an appointment?**

**Will you continue to book appointments online? Why (not)?**

**PART TWO (B) Online appointment booking experiences (People who have *NOT* booked online)**

**Have you heard of online appointment booking?**

**Does your practice offer online appointment booking?**

**How did you find out about it?**

**Prompts:**

- Why don’t you use online appointment booking?
- View on privacy, security, confidentiality?
- Technical issues?

**Would you use online appointment booking in future?**

**What would make you more/less likely to use it?**

**PART THREE – General views**

**What are your views of online appointment booking, in general?**

Prompts:

- What influences your decision to use it rather than other methods?
- How does it fit into your healthcare?
- View on privacy, security, confidentiality?
- What would make it easier to use.

**Do you think online appointment booking has worked well at your GP practice? Why?**

**What do you think is the best booking system for GP surgeries?**
